# Endoscopic ultrasound-directed transenteric retrograde cholangiopancreatography using a new slim scope with an operative working channel

**DOI:** 10.1055/a-2598-3729

**Published:** 2025-06-13

**Authors:** Marco Spadaccini, Alessandro Fugazza, Alessandro De Marco, Matteo Colombo, Vincenzo Craviotto, Cesare Hassan, Alessandro Repici

**Affiliations:** 1 9268 Division of Gastroenterology and Digestive Endoscopy, IRCCS Humanitas Research Hospital, Rozzano, Italy; 2 437807 Department of Biomedical Sciences, Humanitas University, Pieve Emanuele, Milan, Italy

A 79-year-old man, with a history of total gastrectomy with esophagojejunostomy on a Roux-en-Y loop for T2N0 adenocarcinoma, was admitted because of abdominal pain and elevated levels on his liver function tests. A computed tomography (CT) scan showed a stone in the common bile duct (CBD). An entero-endoscopic retrograde cholangiopancreatography was attempted with a long pediatric scope using the underwater cap-assisted technique 1 , but we failed to reached the papillary region owing to the length of the biliary loop. A 7-Fr endoscopic catheter was advanced through the scope back to the proximal jejunum and left in place to facilitate the next steps 2 . Using a linear operative echoendoscope, we identified the duodenal loop, but were then unable to distend it by injecting contrast and fluid through the endoscopic catheter ( Fig. 1 ), so the direct needle-puncture technique was instead used to distend the duodenal loop 3 4 . Once the target loop was sufficiently distended, an entero-enteric anastomosis was created by placing a 15 × 10-mm lumen-apposing metal stent (LAMS) 5 ( Fig. 2 ).

**Fig. 1:**
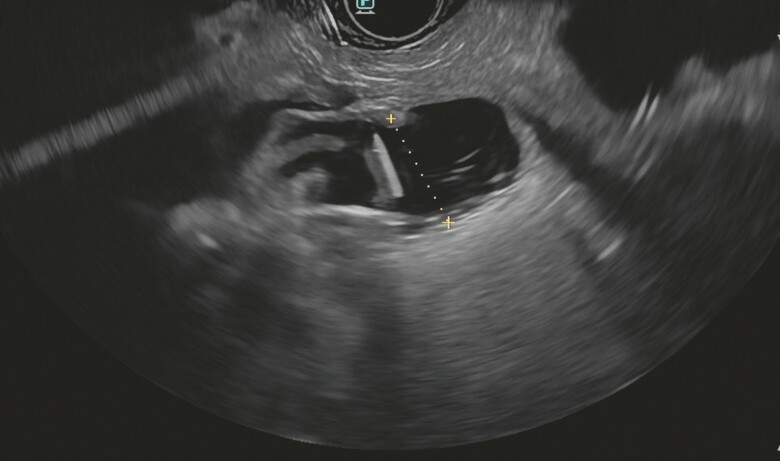
Endoscopic ultrasound image showing the instillation of contrast and saline with methylene blue via a nasojejunostomy tube at the level of the efferent loop of the anastomosis, with correct positioning being confirmed by the aspiration of methylene blue with a 19G needle.

**Fig. 2:**
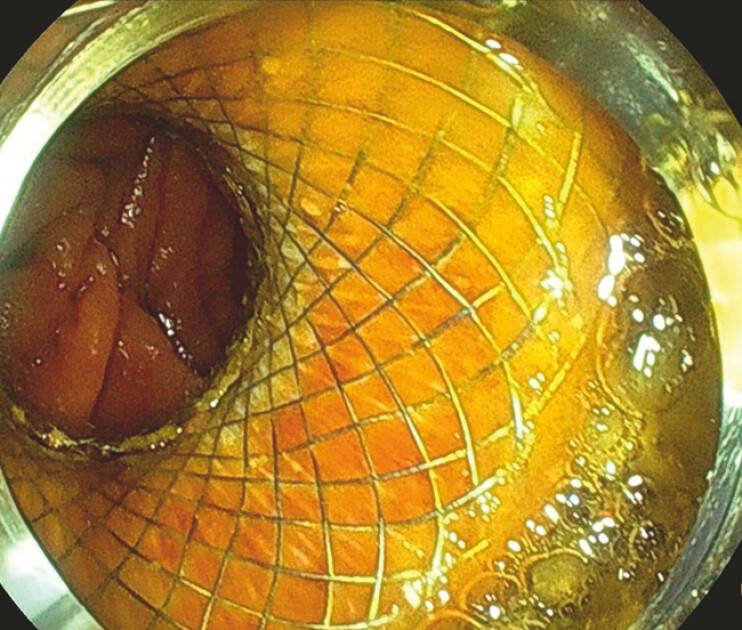
Endoscopic image showing the lumen-apposing metal stent (15 × 10 mm) that was placed to create the entero-enteric anastomosis.

The patient was discharged on the following day, then readmitted 2 weeks after the index procedure, when a new slim gastroscope with a 7.9-mm outer diameter and a therapeutic working channel of 3.2 mm was used to reach the papillary region through the LAMS. Using a catheter and hydrophilic guidewire ( Fig. 3 ), we were able to selectively cannulate the CBD. Pneumatic dilation of the papilla was performed ( Fig. 4 ), and complete clearance of the biliary tract was achieved using an extraction balloon (9–12 mm) ( Fig. 5 ; Video 1 ). The LAMS was subsequently removed 1 month later without any adverse events.

**Fig. 3:**
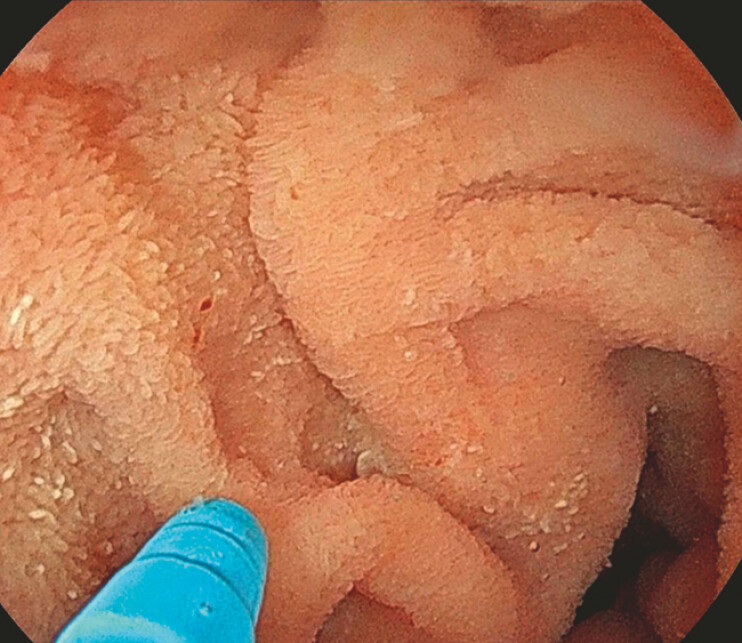
Fluoroscopic image showing cannulation of the bile duct using a forward-viewing scope with a catheter and hydrophilic guidewire.

**Fig. 4:**
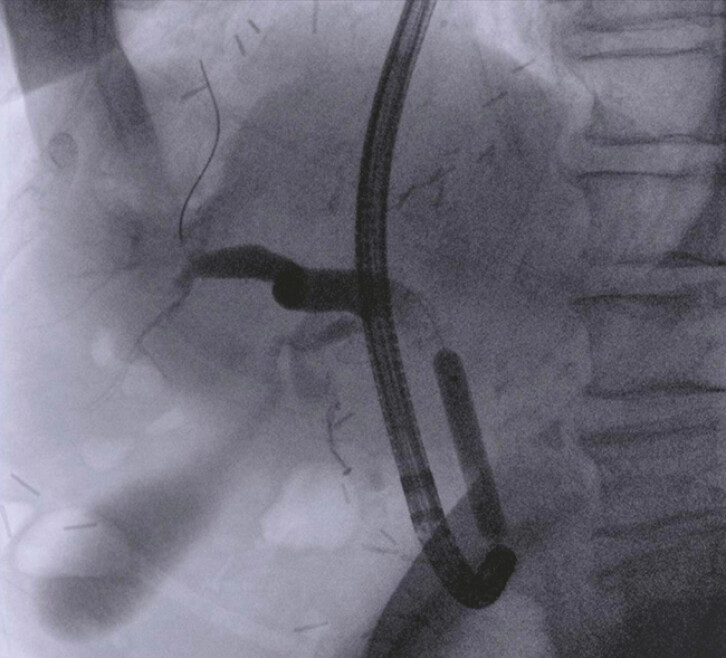
Endoscopic image showing pneumatic dilation of the papilla up to 9 mm using an extraction balloon (CRE).

**Fig. 5:**
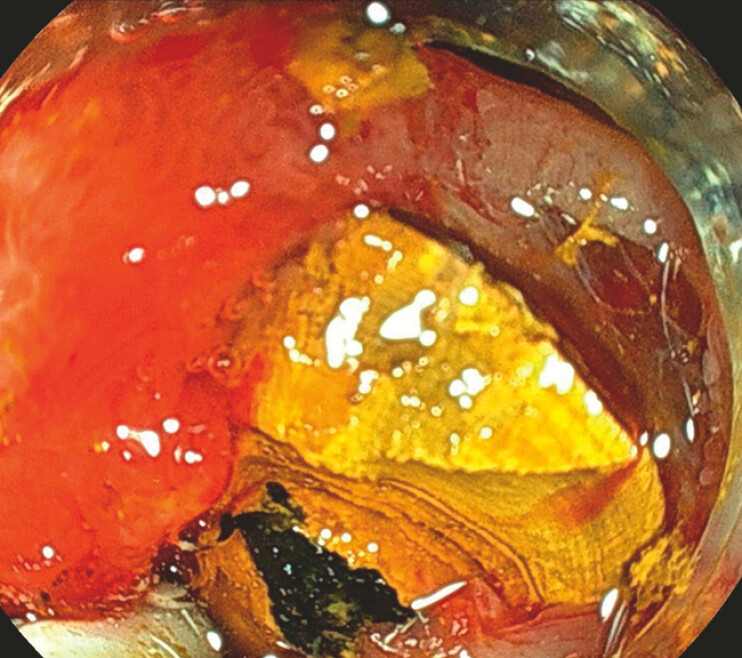
Fluoroscopic image showing clearance of the bile duct using an extraction balloon (9–12 mm).

Endoscopic ultrasound-directed transenteric endoscopic retrograde cholangiopancreatography is performed by placing a lumen-apposing metal stent (15 × 10 mm) and using a forward-viewing scope to perform pneumatic dilation of the papilla up to 9 mm and clearance of the bile duct with an extraction balloon.Video 1

Endoscopy_UCTN_Code_TTT_1AS_2AH

## Notes

Endoscopy E-Videos https://eref.thieme.de/e-videos
*E-Videos* is an open access online section of the journal *Endoscopy* , reporting on interesting cases and new techniques in gastroenterological endoscopy. All papers include a high-quality video and are published with a Creative Commons CC-BY license. Endoscopy E-Videos qualify for HINARI discounts and waivers and eligibility is automatically checked during the submission process. We grant 100% waivers to articles whose corresponding authors are based in Group A countries and 50% waivers to those who are based in Group B countries as classified by Research4Life (see: https://www.research4life.org/access/eligibility/ ). This section has its own submission website at https://mc.manuscriptcentral.com/e-videos .

## References

[1] Fugazza A Anderloni A Paduano D Underwater cap-assisted endoscopic retrograde cholangiopancreatography in patients with surgically altered anatomy: a pilot study Endoscopy 2021 53 927 931 33197940 10.1055/a-1311-9779

[2] Spadaccini M Giacchetto CM Fiacca M Endoscopic biliary drainage in surgically altered anatomy Diagnostics (Basel) 2023 13 3623 10.3390/diagnostics13243623 38132207 PMC10742737

[3] Khashab MA Endoscopic ultrasound-directed transenteric ERCP (EDEE) in patients with postsurgical anatomy – novel but challenging Endoscopy 2019 51 1119 1120 10.1055/a-0958-2323 31775166

[4] Trieu JA Baron TH EUS-guided gastroenterostomy using direct needle-puncture technique VideoGIE 2023 9 164 168 10.1016/j.vgie.2023.10.014 38482479 PMC10927606

[5] Mutignani M Forti E Larghi A Endoscopic entero-enteral bypass: an effective new approach to the treatment of postsurgical complications of hepaticojejunostomy Endoscopy 2019 5 1146 1150 10.1055/a-0914-2855 31163496

